# Supplementary material for: Neural Topology Optimization Via Active Learning for Efficient Channel Design in Turbulent Mass Transfer
Source: Adv Sci (Weinh). 2025 Jul 13;12(37):e08386. doi: 10.1002/advs.202508386 (PMC12499509; doi:10.1002/advs.202508386)
Supplement: Supplementary file 1 — Supporting Information [file ADVS-12-e08386-s001.pdf]

ADVANCED SCIENCE
Open Access

Supporting Information

for *Adv. Sci.*, DOI 10.1002/advs.202508386

Neural Topology Optimization Via Active Learning for Efficient Channel Design in Turbulent Mass Transfer

Chenhui Kou, Yuhui Yin, Min Zhu, Shengkun Jia, Yiqing Luo, Xigang Yuan* and Lu Lu**

Supplementary Information for

Neural topology optimization via active learning for efficient channel design in turbulent mass transfer

Chenhui Kou[a,b,c], Yuhui Yin[a,d], Min Zhu[c], Shengkun Jia[a,*], Yiqing Luo[a], Xigang Yuan[a,*], and Lu Lu[c,*]

[a]School of Chemical Engineering and Technology, State Key Laboratory of Chemical Engineering, Tianjin University, Tianjin 300072, China
[b]College of Chemistry & Chemical Engineering, Yantai University, Yantai 264005, China
[c]Department of Statistics and Data Science, Yale University, New Haven, CT 06511, USA
[d]Department of Chemical Engineering, University College London, London WC1E 7JE, UK
[*]Corresponding author. Email: jiask@tju.edu.cn, yuanxg@tju.edu.cn, lu.lu@yale.edu

S1 Initial dataset

Training dataset. Since the pressure distribution is more sensitive to variations in the topology structure than the concentration distribution, we use a larger dataset to train the $\mathcal{G}_P$ neural operator than $\mathcal{G}_C$. Specifically, we randomly generate 891 topological structures, every 89 structures corresponding to one value of inlet velocity $v \in \{0.1, 0.2, \ldots, 0.9\}$ m/s. The CFD equations are used to solve the pressure distributions, yielding 891 data for training the $\mathcal{G}_P$ neural operator. Then, 351 cases, every 39 structures corresponding to one value of inlet velocity $v \in \{0.1, 0.2, \ldots, 0.9\}$ m/s, are randomly selected to solve the CMT equations and obtain concentration distributions, forming the training dataset for the $\mathcal{G}_C$ neural operator. The 9 cases of the smooth channel ($\gamma(x, y) \equiv 1$) with 9 inlet velocities are also included in the training dataset for both $\mathcal{G}_P$ and $\mathcal{G}_C$. In total, the training dataset for the $\mathcal{G}_P$ neural operator consists of 900 cases, and the training set for the $\mathcal{G}_C$ neural operator consists of 360 cases.

Test dataset. We randomly generate 18 topological structures, every two structures corresponding to one value of $v \in \{0.1, 0.2, \ldots, 0.9\}$ m/s. Numerical methods were employed to solve the CMT equation system and obtain the corresponding pressure and concentration distributions. These datasets serve as the test datasets for the $\mathcal{G}_P$ and $\mathcal{G}_C$ neural operators.

Interpolation dataset. To evaluate the predictive accuracy of the neural operators for pressure and concentration distributions at $v \notin \{0.1, 0.2, \ldots, 0.9\}$ m/s, we randomly generate 16 topological structures, every two structures corresponding to one value of $v \in \{0.15, 0.25, \ldots, 0.85\}$ m/s. This interpolation dataset tests the neural operators' ability to interpolate velocity boundary conditions within the training range.

Extrapolation dataset. To evaluate the neural operators' ability to extrapolate velocity boundary conditions outside the training range at $v \notin [0.1, 0.9]$ m/s, we randomly generate 4 topological structures, every two structures corresponding to one value of $v \in \{0.05, 0.95\}$ m/s.

S2 Validation of neural operators

To obtain the pre-trained neural operators in the TO framework, the $\mathcal{G}_P$ and $\mathcal{G}_C$ neural operators were trained using the initial training dataset. The test, interpolation, and extrapolation datasets were then used to validate the generalization ability of the neural operators. Here, we visualize the ground truth, $\mathcal{G}_P/\mathcal{G}_C$ network prediction, and error for one case from each of the three datasets (Fig. S1). The predictions of the neural operators are in good agreement with the reference solutions. This demonstrates that the neural operator achieves high prediction accuracy across different topological structures and also exhibits predictive capability for cases with interpolated or extrapolated inlet velocities.

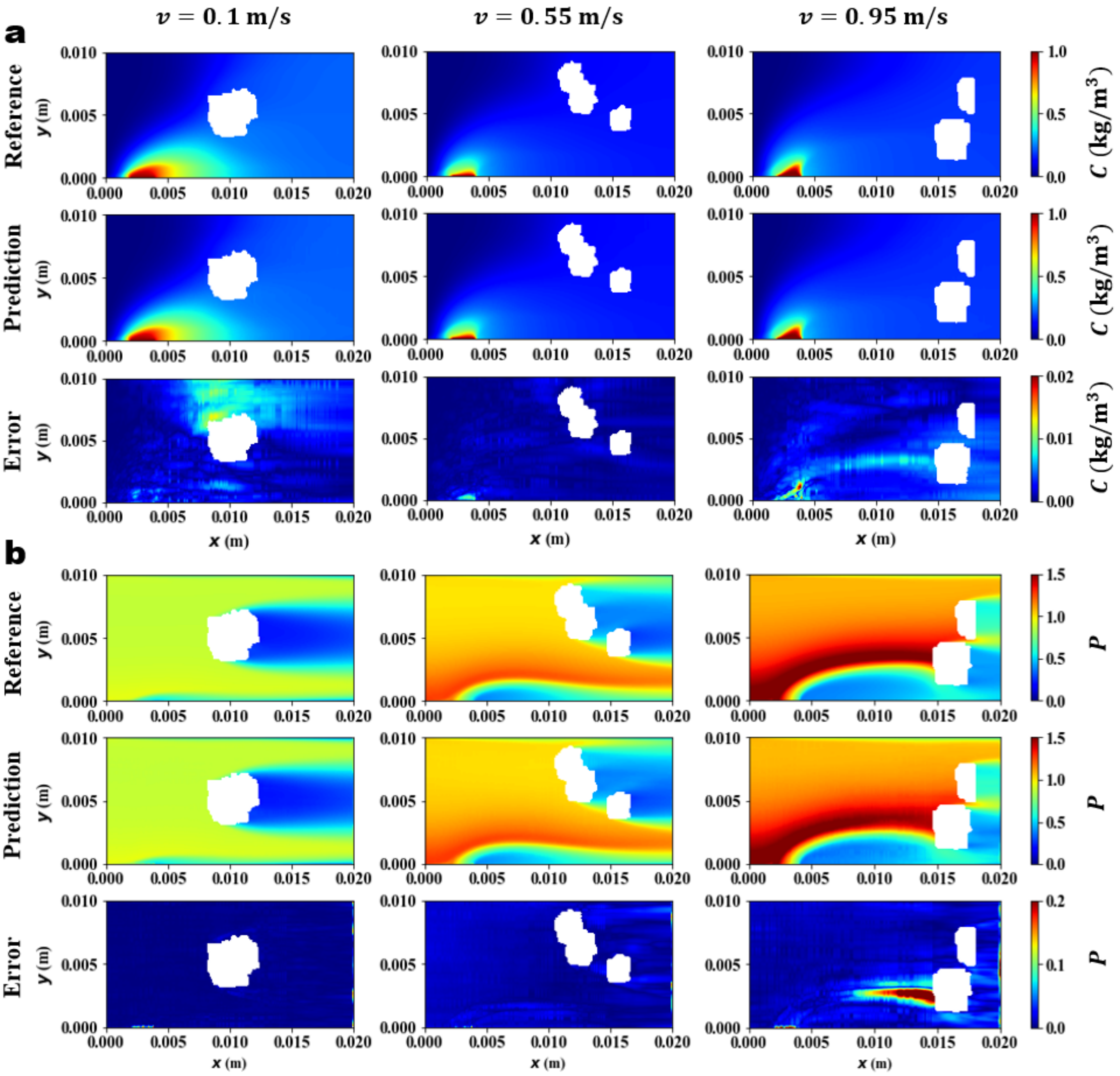

Figure S1: **Validation of the neural operator predictions.** **a**, $\mathcal{G}_C$ results. **b**, $\mathcal{G}_P$ results. Test dataset example with $v = 0.1\,\mathrm{m/s}$; interpolation dataset example with $v = 0.55\,\mathrm{m/s}$; and extrapolation dataset example with $v = 0.95\,\mathrm{m/s}$.

S3 Training procedure

Here, we show the losses during network training, including the losses of the neural operators (Fig. S2) and the neural topology optimization (Fig. S3).

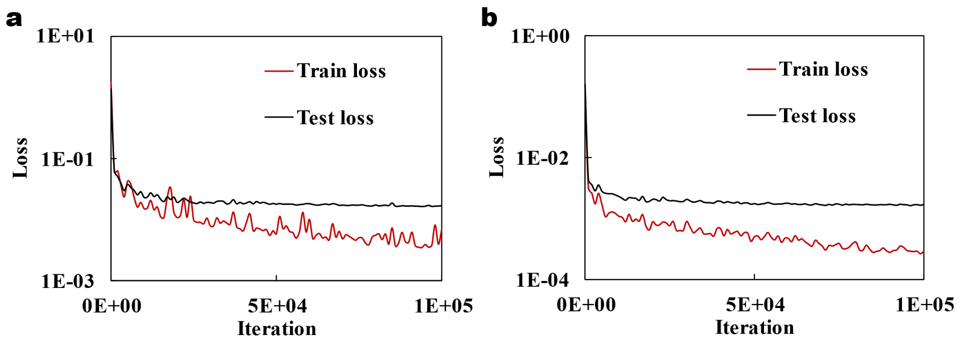

Figure S2: **Training and test losses for neural operators using the initial training dataset.** **a**, $\mathcal{G}_P$ neural operator. **b**, $\mathcal{G}_C$ neural operator.

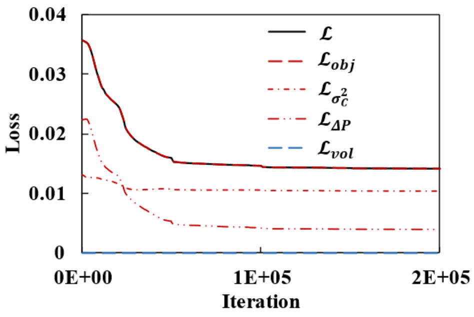

Figure S3: **Training losses of neural topology optimization with** $\omega = 1$**.**

S4 Comparison of three TO results

To validate the effectiveness of the TO algorithm, we use the mechanism model to verify the accuracy of the neural operator's predictions for the optimal topological structures (Table S1). In the active learning-based TO approach, the optimal topological structure is iteratively improved, and we show three topology structure examples of the first active data augmentation in Fig. S4. We also illustrate the evolution of the optimal topological structures under different weights and inlet velocities in Fig. S5.

Table S1: **Mean relative errors of the physical fields (C or P) and objectives (σ_C^2 or ΔP) for the optimized topological structures under three different inlet velocities.**

		Physical field error			Objective error		
Inlet velocity v (m/s)		0.1	0.5	0.9	0.1	0.5	0.9
TO result I	C/σ_C^2	3.281%	2.504%	2.721%	0.547%	0.372%	2.119%
	$P/\Delta P$	7.747%	5.254%	3.855%	16.32%	7.058%	3.577%
TO result II	C/σ_C^2	3.728%	2.506%	2.746%	0.470%	0.350%	2.520%
	$P/\Delta P$	6.040%	2.613%	1.943%	7.286%	1.894%	2.560%
Final TO result	C/σ_C^2	0.515%	0.294%	0.160%	0.259%	0.253%	0.257%
	$P/\Delta P$	0.537%	0.346%	0.546%	0.550%	0.335%	0.480%

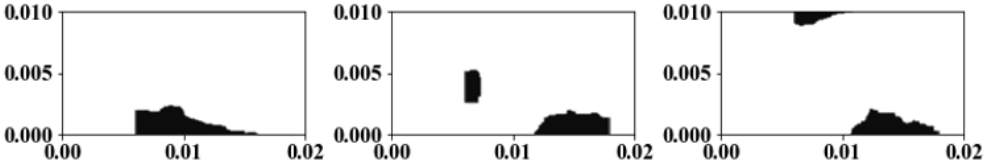

Figure S4: **Examples of topological structures added in the first round of data augmentation.** The black regions represent the solid baffles, where $\gamma = 1$.

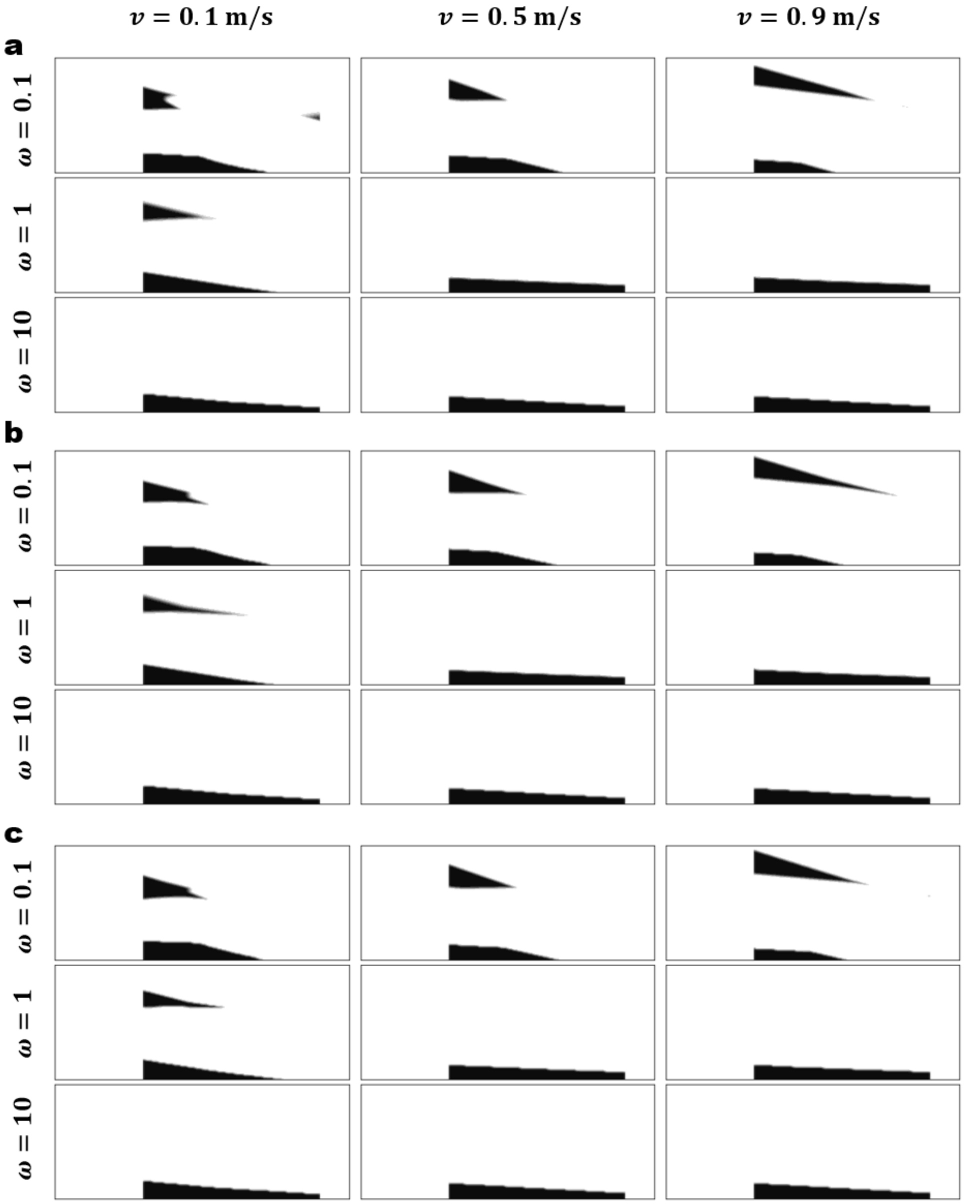

Figure S5: **Optimal topological structures for different inlet velocities and ω.** **a**, TO result I. **b**, TO result II. **c**, Final TO result. The black region represents the solid area, where $\gamma = 1$.

S5 Experimental data

Movies capturing the fluid flow, recorded by a high-speed camera for both the smooth and optimized channel structures, are available at **experiment-data**. The corresponding time-averaged gray images of the smooth and optimized channels are shown in Fig. S6a. We measured the grayscale of standard concentration solutions, establishing a correlation between grayscale and concentration value (Fig. S6b), which is expressed by the decreasing power function: $C = 4.7417\alpha^{-0.7069} - 0.0944$.

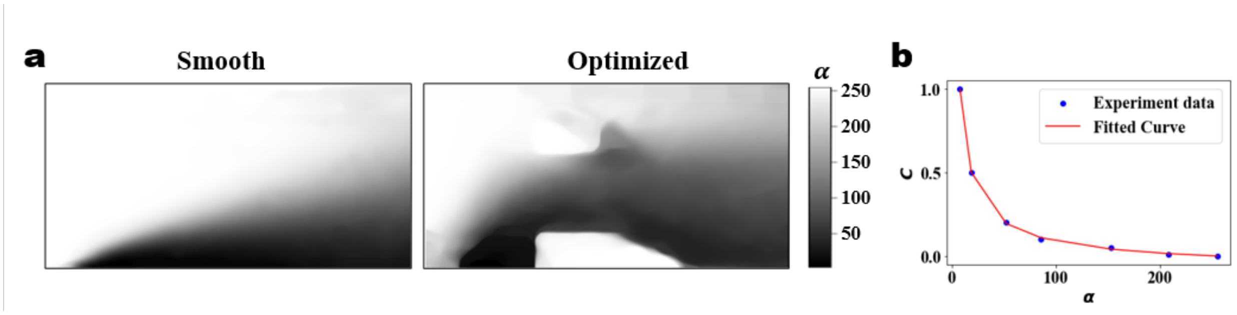

Figure S6: **Experimental data. a**, Time-averaged gray images of the smooth channel and optimized channel. **b**, Gray (α)–concentration (C) calibration curve.
