# Supplementary figures and images for: Neural Topology Optimization Via Active Learning for Efficient Channel Design in Turbulent Mass Transfer
Source: Adv Sci (Weinh). 2025 Jul 13;12(37):e08386. doi: 10.1002/advs.202508386 (PMC12499509; doi:10.1002/advs.202508386)

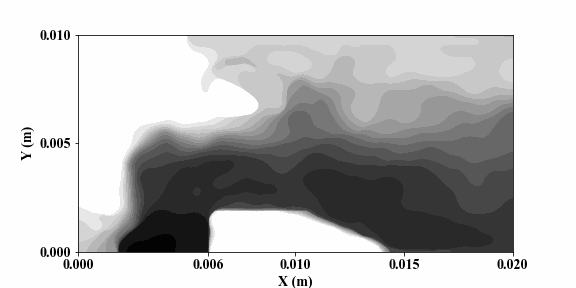

Supplement: Supplementary file 2 — Supplemental Video 1 [file ADVS-12-e08386-s002.gif]

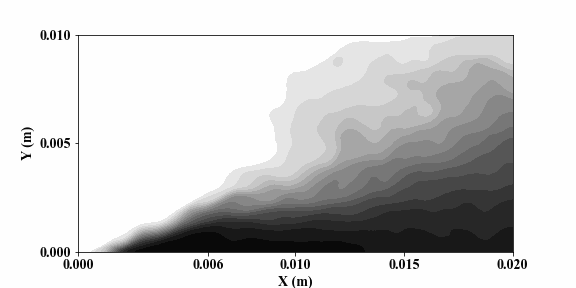

Supplement: Supplementary file 3 — Supplemental Video 2 [file ADVS-12-e08386-s003.gif]
